# Clinical Aspects of Stevens-Johnson Syndrome/Toxic Epidermal Necrolysis With Severe Ocular Complications in Taiwan

**DOI:** 10.3389/fmed.2021.661891

**Published:** 2021-05-12

**Authors:** David Hui-Kang Ma, Tsung-Ying Tsai, Li-Yen Pan, Shin-Yi Chen, Ching-Hsi Hsiao, Lung-Kun Yeh, Hsin-Yuan Tan, Chun-Wei Lu, Chun-Bing Chen, Wen-Hung Chung

**Affiliations:** ^1^Department of Ophthalmology, Chang Gung Memorial Hospital, Taoyuan, Taiwan; ^2^Department of Chinese Medicine, College of Medicine, Chang Gung University, Taoyuan, Taiwan; ^3^Center for Tissue Engineering, Chang Gung Memorial Hospital, Taoyuan, Taiwan; ^4^Department of Ophthalmology, Xiamen Chang Gung Hospital, Xiamen, China; ^5^Department of Ophthalmology, Chang Gung Memorial Hospital, Keelung, Taiwan; ^6^Department of Medicine, College of Medicine, Chang Gung University, Taoyuan, Taiwan; ^7^Department of Dermatology, Chang Gung Memorial Hospital, Taoyuan, Taiwan; ^8^Drug Hypersensitivity Clinical and Research Center, Chang Gung Memorial Hospital, Taoyuan, Taiwan; ^9^Graduate Institute of Clinical Medical Sciences, College of Medicine, Chang Gung University, Taoyuan, Taiwan; ^10^Immune-Oncology Center of Excellence, Chang Gung Memorial Hospital, Taoyuan, Taiwan; ^11^Cancer Vaccine and Immune Cell Therapy Core Laboratory, Chang Gung Memorial Hospital, Taoyuan, Taiwan; ^12^Department of Dermatology, Xiamen Chang Gung Hospital, Xiamen, China

**Keywords:** Stevens-Johnson syndrome, toxic epidermal necrolysis, ocular complication, amniotic membrane transplantation, etanercept, Taiwan

## Abstract

**Purpose:** Over the last decade, there has been tremendous progress in the treatment of Stevens-Johnson syndrome (SJS) and toxic epidermal necrolysis (TEN). To understand whether this has resulted in better ophthalmic outcomes, we aimed to study the incidence of severe ocular complications (SOCs) in the acute and chronic stage among SJS/TEN patients, major causative medications, and therapeutic effect of medical and surgical treatment.

**Methods:** Using electronic medical records review of patients of Chang Gung Memorial Hospital Linkou Branch from 2010 to 2020, 119 patients (236 eyes) received ophthalmic consultation during the acute stage and were retrospectively studied. Sotozono's grading score systems for acute and chronic SJS/TEN were employed for accessing correlation between acute and chronic presentations, the therapeutic effect of systemic etanercept treatment, and outcome of early amniotic membrane transplantation (AMT) performed in patients with severe acute SOCs.

**Results:** There were 46 male and 73 female patients with a mean age of 45.6 ± 22.7 years old (2–90 years), and follow-up time of 408.3 ± 351.0 (116–1,336) days. The numbers of patients with SJS, overlap syndrome, and TEN were 87, 9, and 23, respectively. In total, 109 eyes (55 patients) had acute SOCs, which comprised 46.2% of patients who underwent ophthalmic examination. Antiepileptics were the most common category of culprit drugs causing SOCs in the acute stage. At the end of follow-up, there were 14 eyes (9 patients) with chronic SOCs (5.9%), and non-steroidal anti-inflammatory drugs and cold medicine were the most common causative medications that were associated with severe chronic sequela. The correlation between Sotozono's acute and chronic grading score showed a positive relationship [Spearman's rank correlation coefficient (*r*) = 0.52, *p* < 0.001]. The average chronic grading scores in patients receiving systemic corticosteroid combined with etanercept treatment were significantly lower than those receiving corticosteroid only, Finally, the average chronic grading scores in patients receiving AMT <7 days after onset were significantly lower than those performed beyond 7 days.

**Conclusion:** Our study implies that acute manifestation can be an indicator for chronic sequelae. Additional early etanercept treatment and early AMT showed beneficial effect in reducing chronic ocular sequela.

## Introduction

Stevens-Johnson syndrome/toxic epidermal necrolysis (SJS/TEN) is a potentially life-threatening and vision-impairing immune-mediated disease with an estimated annual incidence of 1–5 per 1,000,000 individuals globally (1). The incidence differs among countries. A study from Korea reported incidences of 3.96–5.03 for SJS and 0.94–1.45 for TEN per million individuals from 2010 to 2013 (2). An incidence rate of 5.76 SJS/TEN cases per million person-years were reported from the UK (database between 1995 and 2013) (3).

Two studies from the US reported similar incidences. The annual SJS spectrum incidence rate reported by White et al. was 12.35 per million people per year (database between 2010 and 2012) (1), and Hsu et al. reported that the mean estimated incidences of SJS, SJS/TEN, and TEN were 9.2, 1.6, and 1.9 per million adults per year, respectively (database between 2010 and 2012) (4).

In Taiwan, utilizing data from the National Health Insurance Research Database (NHIRD) of Taiwan from 2000 to 2008, Syu et al. reported that the overall SJS incidence rate was 3.6 per million people per year (study database between 2000 and 2008) (5). These data may potentially reveal a considerable number of patients affected by ocular complications. Severe ocular complications (SOCs) in chronic SJS/TEN include dry eye, chronic inflammation and neovascularization of the cornea, symblepharon and keratinization of the conjunctiva. If not properly handled, SOCs often result in permanent visual loss.

Because of severe dry eye, SOCs in SJS/TEN are more difficult to treat by surgery; therefore, prevention is the key to reduce the incidence of SOCs in SJS/TEN.

Although Taiwan was the first country to report an intense association of human leukocyte antigen (HLA)-B^*^15:02 and carbamazepine-induced SJS/TEN (6), and the first to implement HLA-B^*^15:02 screening for carbamazepine users (7), reports focusing on ocular manifestations of SJS/TEN from Taiwan are limited. In 2007, Chang et al. reported a total of 207 patients with 213 episodes/attacks of SJS/TEN. The most frequent causative drugs were carbamazepine and allopurinol. Dry eye was the most frequent ocular sequelae identified within 3 months after hospital discharge (17.2%) followed by symblepharon (4.7%) and corneal scarring (4.7%) (8).

Over the last decade, there has been considerable progress not only in diagnostic methods but also in treatment protocols, especially the early application of amniotic membrane transplantation (AMT) (9–12), and it has been shown that the long-term outcomes of AMT to be quite favorable (9, 13). In this article, we aim to study the incidence of SOCs among SJS/TEN patients, major causative medications, the final outcome of affected patients, therapeutic effect of systemic corticosteroids combined with etanercept treatment, and beneficial effect of early AMT.

## Materials and Methods

This retrospective study to collect and analyze electronic medical records of SJS/TEN was approved by the Institutional Review Boards of Chang Gung Medical Foundation (IRB approval No. 202100092B0). All experimental procedures were conducted in accordance with the principles set forth in the Helsinki Declaration.

The list of SJS/TEN patients who were admitted to Chang Gung Memorial Hospital, Linkou Branch from January 2010 to July 2020 was retrieved from the database maintained by the Department of Dermatology. The electronic medical records were retrospectively reviewed, and the recorded data included information on gender, presenting age, causative medications/diseases, and systemic treatments. Regarding disease chronicity, Shanbhag et al. have defined the acute phase as the period between symptom onset of SJS/TEN up to 2 months later; the subacute phase was defined as 2–6 months after symptom onset; and the chronic phase was defined as more than 6 months after symptom onset (14). Ocular manifestations in the acute stage were retrieved from ophthalmic consultation records. To record the severity of ocular involvement at the first time of consultation in the acute stage (within 1 week from the first manifestation), the Sotozono acute stage ocular surface grading score (OSGS) was employed (15). OSGS ranges from grade 0 to 3, with grade 0 indicating no ocular surface involvement, grade 1 indicating mild involvement with conjunctival hyperemia, grade 2 indicating severe involvement with accompanying pseudomembrane formation or ocular surface epithelial defects, and grade 3 indicating very severe involvement with both pseudomembrane formation and ocular surface epithelial defects. The eyes with greater than grade 2 ocular involvement were considered to have acute severe ocular complications (SOCs). The initial VA and ophthalmic medical and surgical treatments were also reviewed. Visual acuity (VA) was evaluated in naked-eye with standard Snellen E chart. Regarding the causative medications, those with acute SOCs (grade 2 and grade 3) were analyzed and compared in percentage.

In the chronic stage (defined as at least 6 months from the first manifestation), ocular involvement was assessed according to the scoring system proposed by Sotozono et al. (16). Complications were broadly defined as corneal, conjunctival, and eyelid complications. There are a total of 13 categories of findings, such as superficial punctate keratopathy (SPK), hyperemia, trichiasis, and symblepharon. Each category scored from 0 to 3, and the maximal score was 39. Treatment in the chronic stage, Schirmer test values and vision were also recorded. Because there was a high portion of missing data for Schirmer test values, the diagnosis of dry eye was made if the patient's Schirmer's test I value (without topical anesthesia) was <10 mm and/or the cornea showed greater than grade 1 SPK. To judge the severity of the ocular manifestation in the chronic stage, the patient's last electronic medical records with a detailed eye examination was used as a reference (Table 1). Patients with Sotozono's chronic stage grading scores 0–5 were considered to have non- or minimal ocular involvement. Patients with scores 6–10 exhibited mild involvement, scores 11–15 indicate moderate involvement, and scores > 16 were considered to have severe involvement.

**Table 1:** Demographics and grading of patients receiving ophthalmic examination in acute and chronic stage of SJS/TEN from 2010 to 2020.

**Demographics/characteristics**	
Number of patients	119
Total eye number	236
Mean age ± SD (range)	45.6 ± 22.7 (2–90)
**Gender, n (%)**	
Male	46 (38.7)
Female	73 (61.3)
**Laterality, n (%)**	
Bilateral	117 (98.3)
Unilateral	2 (1.7)
**Diagnosis, n (%)**	
SJS	87 (73.1)
Overlap syndrome	9 (7.6)
TEN	23 (19.3)
Mean follow-up time (days)	408.3 ± 351.0 (116–1,336)
**Sotozono's acute stage grading scores**, ***n*** **=** **eyes (%)**	
Grade 0	30 (12.7)
Grade 1	97 (41.1)
Grade 2	79 (33.5)
Grade 3	30 (12.7)
Mean OSGS in acute stage (range)	1.46 ± 0.87 (0–3)
**Sotozono's chronic stage grading scores**, ***n*** **=** **eyes (%)**	
0–5	195 (82.6)
6–10	22 (9.3)
11–15	5 (2.1)
>16	14 (5.9)
Mean OSGS in chronic stage (range)	3.54 ± 5.50 (0–33)

*SJS, Stevens-Johnson syndrome; TEN, toxic epidermal necrolysis; OSGS, ocular surface grading score*.

Based on published therapeutic benefits of steroid therapy at disease onset in preventing ocular complications (17), systemic treatment with steroid pulse therapy (1–1.5 mg/kg/day prednisolone by intravenous injection until the skin lesions were healed), intravenous methylprednisolone or hydrocortisone has also been commonly administered to SJS/TEN patients admitted to dermatology wards (17, 18). Based on the conclusion from a randomized and controlled trial from our hospital (2009–2015) (19), additional subcutaneous etanercept (Enbrel; Pfizer) has been prescribed for recalcitrant SJS/TEN patients with progression of blistering or erythema even after methylprednisolone (>1 mg/kg/day) treatment for 3–5 days. To investigate the effect of additional systemic etanercept on long-term ocular outcome, we focused on patients whose medical records showed treatment with intravenous corticosteroids with or without additional subcutaneous etanercept. Demographics of the patients in each group such as age, gender, and distribution of top 5 culprit drugs were compared, and we found no significant difference in these parameters (**Table 5**). Thereafter, the acute and chronic stage severity grading scores were compared between these two groups.

In the acute stage, we used topical balanced salt solution (BSS, Alcon) for lubrication, levofloxain (Cravit, Santen) as a prophylactic antibiotic, Tobradex ointment (Alcon) as topical corticosteroid ointment for eyelid wounds, and preservative-free 0.1% betamethasone (Fusone, AIM Medicine, Taiwan) qid to q3h when conjunctival congestion was evident. Conjunctival pseudomembranes were removed every 2–4 days. If grade 3 and sometimes grade 2 ocular involvement was noted, amniotic membrane (AM) dressing using cryopreserved AM was performed to protect the entire ocular surface. Before 2019, suture-fixated AM dressing was performed (20–22). Since 2020, sutureless AM dressing using cyanoacrylate glue to fixate AM at the lid margin has been adopted (23). This novel technique significantly reduced the time and discomfort of the surgery. To study the influence of timing of AM transplantation (AMT; AM grafting or AM dressing) on the outcome, we compared the Sotozono's chronic grading score of patients who received AMT less than or more than 7 days after onset.

### Statistics

All statistics were calculated using SPSS software version 23.0 for Windows (SPSS, Inc., Chicago, IL, USA). Continuous variables are presented as the mean and standard deviation (SD). Spearman's rank correlation was used to present the association between Sotozono's acute and chronic grading score. Correlation coefficients (ρ) were also calculated. Student's *t*-test was used to compare the difference in demographics, follow-up duration, acute and chronic grading scores between patients treated with or without additional etanercept, and chronic grading scores in patients receiving AMT before or beyond 7 days after onset. Chi-squared test was used to compare distribution of causative medications between patients treated with or without additional etanercept. A *p* < 0.05 was deemed to be statistically significant.

## Results

From January 2010 until July 2020, a total of 294 patients were registered in the Department of Dermatology database. Among them, 119 patients (236 eyes) received ophthalmic consultation during the acute stage and were recruited in this study. There were 46 male and 73 female patients with a mean age of 45.6 +/– 22.7 years old (2–90 years). The numbers (percentages) of patients with SJS, overlap syndrome, and TEN were 87 (73.1%), 9 (7.6%), and 23 (19.3%), respectively. When the number of involved eyes was calculated, using Sotozono's acute OSGS, there were 30 eyes without ocular involvement (grade 0, 12.7%), 97 eyes with mild involvement (grade 1, 41.1%), 79 eyes with severe involvement (grade 2, 33.5%), and 30 eyes with the most severe involvement (grade 3, 12.7%). Given that patients with greater than grade 2 ocular involvement were considered to have SOCs, there were 109 eyes (55 patients) in total with acute SOCs, comprising 46.2% of patients who had ophthalmic examination, and 18.6% of total patients (Table 1). In the chronic stage, when the assessment was done at an averaged 408.3 ± 351.0 (116–1,336) days after onset, there were 195 eyes (82.6%) with Sotozono's chronic grading scores 0–5 (non- or minimal ocular involvement). Twenty-two eyes (9.3%) with scores 6–10 (mild involvement), 5 eyes (2.1%) with scores 11–15 (moderate involvement), and 14 eyes (5.9%) with scores >16 (severe involvement) (Table 1).

Among 67 identifiable drugs that were associated with acute SOCs, antiepileptics (carbamazepine, phenytoin, lamitrogine, etc.) was the category of drugs that were associated with most acute SOCs (*n* = 15, 22.4%) followed by antibiotics (*n* = 12, 17.9%), allopurinol (*n* = 8, 11.9%), non-steroidal anti-inflammatory drug (NSAID; *n* = 7, 10.4%), and sulfa drugs (*n* = 6, 9.0%) (Table 2). In terms of a single drug, allopurinol was the drug that was associated with most acute SOCs. In 14 eyes of the 9 patients with chronic SOCs, NSAID and cold medicine were the most common causative medications that were associated with chronic SOCs (44.4%, Table 2).

**Table 2:** Top 5 causative drug categories responsible for acute and chronic SOCs in SJS/TEN patients.

**Name of causative drugs**	**No. of patients (%)**
**Acute SOCs**	***N*** ****=**** **67**
**Antiepileptics**	**15 (22.4)**
Phenytoin	2 (3.0)
Carbamazepine	4 (6.0)
Oxcarbazepine	5 (7.5)
Lamotrigine	2 (3.0)
Zonisamide	2 (3.0)
**Antibiotics**	**12 (17.9)**
Cephalexin	1 (1.5)
Cefuroxime	1 (1.5)
Ceftazidime	1 (1.5)
Ceftriaxone	1 (1.5)
Norfloxacin	1 (1.5)
Levofloxacin	1 (1.5)
Moxifloxacin	1 (1.5)
Amoxicillin	3 (4.5)
Vancomycin	2 (3.0)
**Allopurinol**	**8 (11.9)**
**NSAIDs**	**7 (10.4)**
Diclofenac	3 (4.5)
Ibuprofen	1 (1.5)
Mefenamic acid	3 (4.5)
**Sulfa drugs**	**6 (9.0)**
Sulfasalazine	3 (4.5)
Sulfonamide	3 (4.5)
**Miscellaneous**	**19 (28.4)**
**Chronic SOCs**	***N*** ****=**** **9**
**NSAIDs**	**4 (44.4)**
Diclofenac	2 (22.2)
Mefenamic acid	1 (11.1)
Cold medicine	19 (11.1)
**Anti-epileptics**	**2 (22.2)**
Carbamazepine	1 (11.1)
Lamotrigine	1 (11.1)
**Antibiotics**	
Cefuroxime	1 (11.1)
**Sulfa drugs**	
Sulfasalazine	1 (11.1)
**Tenofovir**	**1 (11.1)**

*SOCs, Severe ocular complications*.

*NSAIDs, Non-steroidal anti-inflammatory drugs*.

**Figure 1:**
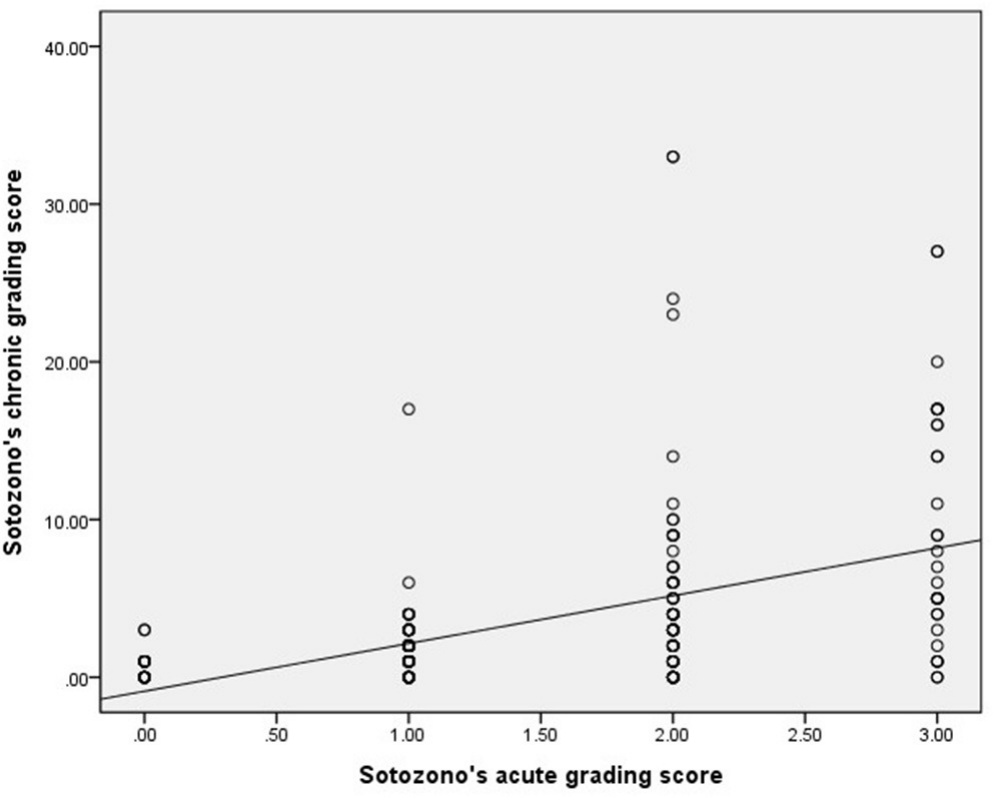
Correlations between Sotozono's acute (15) and chronic (16) stage grading score of SJS/TEN patients evaluated during the acute and chronic stage, respectively.

The average grading score for all patients in the acute stage was 1.46 ± 0.87. The value in the chronic stage (at the end of follow-up) was 3.54 ± 5.50. For patients whose grading score ranged from 0 to 3 during the acute stage, their corresponding chronic stage grading scores were 0.67 ± 0.80, 1.62 ± 1.99, 4.70 ± 6.38, and 9.60 ± 7.73, respectively. The correlation between Sotozono's acute and chronic grading score showed a moderately correlated relationship [Spearman's rank correlation coefficient (ρ) = 0.52, *p* < 0.001] (Figure 1). This finding implies that patients who presented with more severe acute ocular manifestations tended to suffer from sequelae in the chronic stage.

Judging from Sotozono's chronic stage ocular complication category, hyperemia was the most common chronic stage ocular complication, followed by SPK, meibomian gland dysfunction, mucocutaneous junction involvement, and trichiasis. Judged from the Schirmer test value and the presence of corneal staining, the incidence of dry eye in the chronic stage was estimated to be 49.2% (116 eyes) (Table 3).

**Table 3:** Ocular manifestations in chronic stage of SJS/TEN patients.

**Characteristic**	**Number of eye**	**Percentage (%)**
**Corneal complication**		
Superficial punctate keratitis	117	49.58
Epithelium defect	29	12.29
Loss of POV^*^	9	3.81
Conjunctivalization	13	5.51
Neovasculization	22	9.32
Opacification	19	8.05
Keratinization	14	5.93
**Conjunctival complication**		
Hyperemia	122	51.69
Symblepharon	8	3.39
**Eyelid complication**		
Tichiasis	49	20.76
MC^*^ junction involvement	62	26.27
Meibomian gland involvement	90	38.14
Punctal damage	7	2.97

* *POV, Palisades of Vogt; MC, Mucocutaneous*.

When best corrected vision in the chronic stage was measured, data were available from 174 eyes. Among them, 112 eyes (64.4%) had vision better than 20/40, and 43 eyes (24.7%) had vision between 20/40 and 20/200. Nine eyes (5.2%) had vision between 20/200 and 20/2,000, and 10 eyes (5.7%) had vision worse than 20/2,000.

To study the effect of additional systemic etanercept on long-term ocular manifestations, there was no significant difference in gender, age, distribution of culprit drugs, and follow-up time between patients receiving corticosteroid treatment only and patients receiving corticosteroid plus etanercept treatment (Table 4). The average acute stage score for patients who received additional systemic etanercept was 1.32 ± 0.76 (0–3). The value for patients who received systemic corticosteroid only treatment was 1.49 ± 0.90 (0–3; *p* = 0.171). In the chronic stage, the average score for additional etanercept treated patients was 1.64 ± 2.47 (0–14), which was significantly lower than the score for corticosteroid only treated patients (3.95 ± 5.76; range 0–33, *p* < 0.001) (Table 4).

**Table 4:** Comparison between corticosteroid with or without additional etanercept treatment on Sotozono's acute and chronic stage grading score.

	**Corticosteroid +**	**Corticosteroid**	***p-*value**
	**etanercept**	**only**	
Number (patient/eye)	31/62	82/164	
Gender (M/F)	15/16	32/50	0.126
Age (years old)	48.00 ± 18.72	45.79 ± 24.06	0.601
Mean follow up time (days)	485.00 ± 502.42	360.82 ± 319.64	0.319
Time to intervention (days)	1.96 ± 1.49	0.78 ± 0.64	0.003
**Major causative drugs**			
Anti-epileptics	24 (38.71%)	48 (29.27%)	0.264
Antibiotics	11 (17.74%)	26 (15.58%)	
Allopurinol	6 (9.68%)	10 (6.10%)	
NSAID	6 (9.68%)	26 (15.58%)	
Sulfa drugs	2 (3.23%)	18 (10.98%)	
Others	13 (20.97%)	36 (21.95%)	
**Sotozono's grading score**			
**Acute stage**			
Mean	1.323	1.488	0.171
Std. deviation	0.763	0.903	
Std. Error Mean	0.097	0.071	
**Chronic stage**			
Mean	1.645	3.951	<0.001
Std. deviation	2.470	5.765	
Std. error mean	0.314	0.450	

Finally, for surgical treatment, during the acute stage (<2 months after onset), 7 patients (13 eyes) received AM grafting (AMG) and 7 patients (14 eyes) received AM dressing (AMD) at an averaged 8.15 ± 4.5 days and 4.93 ± 4.6 days, respectively, after onset. During the chronic stage, seven patients (12 eyes) received oral mucosal transplantation to correct lid margin keratinization, 4 patients (7 eyes) received punctal occlusion to treat dry eye, and 2 patients (4 eyes) received correction of entropion (Table 5). When we compare the Sotozono's chronic stage grading scores in patients receiving AM transplantation (AMG + AMD) less than or more than 7 days after onset, we found that in AMG group and combined AMG and AMD group, the scores were significantly lower when the operation was performed <7 days (2.17 ± 2.63 vs. 15.6 ± 12.94, *p* = 0.031; 6.29 ± 4.69 vs. 13.60 ± 11.22, *p* = 0.025; Chi-squared test). This implies that patients receiving earlier AM transplantation were associated with less severe chronic SOCs (Table 5).

**Table 5A:** Surgical intervention in the acute and the chronic stage of SJS/TEN.

**Type of**	**Patient**	**Eye**	**Time to**
**surgery**	**(%)**	**(%)**	**intervention (days)**
**Acute stage**			
AMG*	7 (5.88)	13 (5.51)	8.15 ± 4.5 (3–15)
AMD*	7 (5.88)	14 (5.93)	4.93 ± 4.6 (0–14)
**Chronic stage**			
OMT*	7 (5.88)	12 (5.08)	
Punctum suture	4 (3.36)	7 (2.97)	
Correction of entropion	2 (1.68)	4 (1.69)	

**Table 5B:** Comparison of Sotozono's chronic stage grading score in patients receiving AM transplantation more than or < 7 days after onset.

**Type of surgery**	**Onset to intervention**	**Chronic stage**	***p*-value**
	**(in days)**	**grading score**	
AMG	≧7 day (*n* = 7)	15.6 ± 12.94 (4~33)	0.031
	<7 day (*n* = 6)	2.17 ± 2.63 (0~6)	
AMD	≧7 day (*n* = 3)	8.33 ± 2.08 (6~10)	0.904
	<7 day (*n* = 11)	8.55 ± 3.98 (5~16)	
AMG + AMD	≧7 day (*n* = 10)	13.60 ± 11.22 (4~33)	0.025
	<7 day (*n* = 17)	6.29 ± 4.69 (0~16)	

*AMG, Amniotic membrane graft; *AMD, Amniotic membrane dressing; *OMT, Oral mucosal transplantation.

## Discussion

In multiple-country epidemiological studies of medication risk related to SJS/TEN in Asian populations (1998–2017), Wang et al. reported that antiepileptic drugs/antipsychotics (53%) were the most common category of drugs that caused SJS/TEN followed by antibiotics/antiviral agents (20%), allopurinol (19%), and NSAIDs (4%) (24). In Taiwan, Chang et al. previously reported that carbamazepine was the most common medication that caused SJS/TEN followed by allopurinol and phenytoin (8). With the ground-breaking finding that the human leukocyte antigen HLA-B^*^15:02 was intensively associated with carbamazepine-induced SJS/TEN in Han Chinese individuals (6), subsequent government health insurance covering HLA-B^*^15:02 screening dramatically reduced the incidence of carbamazepine-induced SJS/TEN (7). When all registered patients within the last 10 years were reviewed, antiepileptics were still the most common category of drugs that caused acute SOCs followed by antibiotics and allopurinol. However, allopurinol was the most common single culprit drug that caused acute SOC followed by oxcarbazepine and carbamazepine. Recently, cold medicine (antipyretic or analgesic but not antibiotics a patient takes when catching cold) was thought to be the most common drug that induces SOCs in chronic SJS/TEN (25–29). We also had similar observations in our recent study based on patients from outpatient clinics, and identified HLA B^*^0207 to be associated with cold medicine-induced SJS/TEN with SOCs (30). In this study, NSAID and cold medicine were found to be the most common causative medications that were associated with chronic SOCs. A significant association was noted between HLA-B^*^15:02 and oxcarbazepine-induced SJS/TEN; however, the incidence and severity were lower than those of carbamazepine-induced STS/TEN (31). On the other hand, although a strong association of HLA-B^*^5801 with allopurinol-induced SJS/TEN has been reported (32, 33), the relatively lower incidence of allopurinol-induced SJS/TEN does not support the implementation of gene screening.

One may think that patients with more severe dermatological involvement also have more severe ocular involvement. However, Morales et al. reported that although ocular damage in the acute stage was more frequent in patients with epidermal detachment >10% of the total body surface area, the SCORTEN value did not correlate with the severity of eye involvement in the acute stage (34). Heng et al. reported that the grading of acute ocular disease severity does not correlate with systemic disease severity but is significantly associated with the time to resolution of ocular involvement in TEN (35). Yip et al. also stressed that the severity of acute ocular disease and abnormal laboratory tests were not found to be significant risk factors for late complications (36).

To identify predictors for the development of chronic ocular complications, Gueudry et al. retrospectively reviewed the records for demographics, cause of the condition, and severity of ocular involvement. They found that (1) Patients with TEN had more frequent but not more severe acute ocular involvement; (2) Dry eye syndrome was the most common late complication; (3) The severity of acute ocular disease was found to be the only significant risk factor for late complications. Although late complications are more frequent in patients with severe initial eye involvement, these complications may also develop in patients without initial ocular symptoms (37). In this study, we found a good correlation between the severity of acute ocular manifestations and chronic ocular sequelae; nevertheless, we also observed that some patients with only minimal ocular involvement in the beginning deteriorated over the years due to insults from chronic inflammation, dry eye, trichiasis and lid margin keratinization. Therefore, we agree with Shanbhag et al. that patients with any acute ocular involvement regardless of severity should be seen by an ophthalmologist for life given that severe and irreversible complications can occur at any time, even decades after acute disease (38).

There are several different grading systems to define the severity of ocular involvement in chronic SJS/TEN (16, 39, 40). We chose to use Sotozono's 2007 grading system because the system includes the most observation features, each with a score range, which facilitates quantitative comparison between different groups (16). We found hyperemia to be the most common chronic stage ocular complication followed by dry eye, SPK, and meibomian gland dysfunction. However, in our hospital, not all acute SJS/TEN patients (294 patients in the last decade) were seen by ophthalmologists. It was only when patients presented with red eye or other ophthalmic symptoms that dermatologists sent out a consultation request (119 patients, 236 eyes in total). This is the major drawback in the study as this might overestimate the SOC rate given that many non-consulted patients were in fact patients without any ocular involvement. For example, the percentages of patients with acute SOCs, chronic SOCs, and late severe vision impairment (< 20/200) were 46.2, 5.9, and 10.9%, respectively, among consulted patients, but the value could be as low as 18.7, 2.4, and 3.4%, respectively, if non-examined patients were all considered free of eye involvement. This projected value would be very similar to the report by Power et al. which indicates that 16% of patients with SJS-spectrum will experience severe ocular involvement (41), and the report by Shanbhag et al. which indicates that only 3% eyes had vision worse than 20/200 after aggressive treatment (14).

Previously, a high portion of SJS/TEN patients often suffered from chronic debilitating sequelae. In 2007, Sotozono et al. reported that greater than half of their patients had final vision worse than 20/200 (74 eyes, 53.6%) with an average chronic SJS grading score of 25.66 (16). With the awareness that early and aggressive intervention in the acute stage is key to prevent long-term complications, a specific protocol for acute ocular care in SJS/TEN, including aggressive use of AMT (22, 23), was instituted at Massachusetts Eye Infirmary in January 2008, which was highly successful in reducing corneal blindness and severe vision-threatening complications (14). In the report of Shanbhag et al. after adapting the protocol, only 3% (2/78) of eyes had vision worse than 20/200 in contrast to 50% (9/18) before the protocol (14). In this study, systemic corticosteroids were administered to all but 5 patients in the acute stage, and 27 of the 30 eyes with acute grade 3 involvement received either AMG or AMD. We believe that in the last decade, aggressive systemic immunosuppressives and early AM transplantation have already contributed to reduced long-term ocular complications. In this study, the average chronic grading scores in patients receiving AMT <7 days after onset were significantly lower than those performed beyond 7 days, suggesting the beneficial effect of early AMT in reducing chronic SOCs (9, 13).

In addition to systemic corticosteroids (17, 39), other immunomodulatory medications, such as intravenous immunoglobulin (IVIG) (39, 42) and cyclosporine A (43, 44), have been used to treat acute SJS/TEN. Although IVIG was found to be ineffective, the use of cyclosporine may offer a greater mortality benefit (45) in the treatment of SJS/TEN, and cyclosporine and glucocorticosteroids were shown to be the more promising systemic immunomodulating therapies (43). Recently, our dermatology colleagues reported the beneficial effect of systemic etanercept treatment in promoting epidermal regeneration and reducing the incidence of gastrointestinal hemorrhage and mortality in acute SJS/TEN in a randomized, controlled study (19). The use of subcutaneous etanercept is generally safe, but we need to pay attention to whether there is tuberculosis, hepatitis B, hepatitis C, and serious infections before the injection. In the present study, we also found that patients receiving additional systemic etanercept exhibited better final vision and significantly lower chronic SJS/TEN grading scores. This encourages us to conduct a study to trace the long-term ocular condition in the original cohort of patients (manuscript in preparation).

In summary, we found that patients with severe acute ocular manifestations in SJS/TEN tend to suffer from chronic ocular involvement. Antiepileptics were the category of drugs that caused most acute SOCs, whilst NSAIDs and cold medicines were associated with most chronic SOCs. With aggressive medical and surgical treatment, eyes suffering from chronic complications of SJS/TEN have decreased in the last decade. Finally, systemic etanercept given in the acute stage showed promise in reducing long-term ocular morbidity; however, the efficacy and long-term benefit still await further investigation.

## Data Availability Statement

The original contributions presented in the study are included in the article/supplementary material, further inquiries can be directed to the corresponding author/s.

## Ethics Statement

The studies involving human participants were reviewed and approved by Institutional Review Boards of Chang Gung Medical Foundation. Written informed consent from the participants' legal guardian/next of kin was not required to participate in this study in accordance with the national legislation and the institutional requirements.

## Author Contributions

DM: writing of the manuscript, patient care and surgery. T-YT: review of electronic medical records, statistical analysis, and making of tables and figure. L-YP: review of electronic medical records, statistical analysis, and making of tables. S-YC: patient care and surgery. C-HH, H-YT, and L-KY: ophthalmic consultation. C-WL: dermatological care of patients. C-BC: dermatological care of patients and provides patient list. W-HC: dermatological care of patients and inventor of systemic etanercept treatment. All authors contributed to the article and approved the submitted version.

## Conflict of Interest

The authors declare that the research was conducted in the absence of any commercial or financial relationships that could be construed as a potential conflict of interest. The handling Editor declared a past collaboration with several of the authors DM, W-HC, and S-YC.
